# Editorial: Development of Circadian Clock Functions

**DOI:** 10.3389/fnins.2021.735007

**Published:** 2021-08-09

**Authors:** Jihwan Myung, Takahiro J. Nakamura, Jeff R. Jones, Rae Silver, Daisuke Ono

**Affiliations:** ^1^Graduate Institute of Mind, Brain, and Consciousness, Taipei Medical University, Taipei, Taiwan; ^2^Brain and Consciousness Research Center, Taipei Medical University-Shuang Ho Hospital, Ministry of Health and Welfare, Taipei, Taiwan; ^3^Laboratory of Animal Physiology, School of Agriculture, Meiji University, Tokyo, Japan; ^4^Department of Biology, Washington University in St Louis, St. Louis, MO, United States; ^5^Department of Psychology, Columbia University, New York, NY, United States; ^6^Department of Neuroscience and Behavior, Barnard College of Columbia University, New York, NY, United States; ^7^Department of Neuroscience II, Research Institute of Environmental Medicine, Nagoya University, Nagoya, Japan; ^8^Department of Neural Regulation, Nagoya University Graduate School of Medicine, Nagoya, Japan

**Keywords:** circadian rhythm, development, plasticity, neural network, aging

The temporal organization of physiological functions such as sleep/wakefulness or body temperature are regulated by the circadian clock. This intrinsic clock starts ticking in the embryo, matures during development, and is attenuated in the elderly. This is illustrated by weakening synchrony, entrainment, and outputs of cellular circadian rhythms in the central circadian clock, located in the suprachiasmatic nucleus (SCN) of the hypothalamus. The age-related diminution can contribute to susceptibility to diseases, such as sleep disorders, infertility, diabetes, and mental disorders. Over the course of our lives, a variety of internal and external factors come into play, under the influence of the circadian clock. The inherent developmental plasticity of the circadian system, the focus of the present issue, is critical for the establishment of normal bodily functions and their adaptation to the changing environment on earth.

At the single-cell level, the circadian clock is a transcriptional-transcriptional feedback loop (TTFL) that underlies the 24-h rhythmicity in gene expression, which is maintained even under challenges on some of the feedback components of the loop (Chiou et al. in this collection). Chiou et al. created sextuple knockout (*Cry1/2*^−/−^; *Per1/2*^−/−^; *Nr1d1/2*^−/−^) cell lines using the CRISPR/Cas9 system and found that serum-shock inhibited CLOCK-BMAL1 in the absence of CRY, PER, and NR1D proteins. The finding lets us speculate on the TTFL during early development, where the stoichiometry of clock components is different from adults (Li and Davis, 2005). During development, the molecular components required for the emergence of the molecular circadian clock are produced *de novo* in embryonic stem cells (Yagita et al., 2010), causing time-delayed ontogenesis of tissue-level circadian clocks (Sládek et al., 2007) including the networks of the central circadian clock in the SCN (Cheng and Cheng in this collection). The network-level development confers both robustness and coding complexity to circadian clocks. Coupling among circadian clocks not only synchronizes component cellular oscillators but also provides robustness against genetic perturbations (Liu et al., 2007). The characteristics of the coupled cells [such as the peptidergic expression profile (Romero and Silver, 1990)] and the nature of coupling itself, changes through development, and can allow for unexpected circadian oscillations in the perinatal network (Ono et al., 2013; Tokuda et al., 2015). Such early developmental processes may be comparable to excessive positive coupling and subsequent pruning found in other brain regions. In the SCN, network fine-tuning occurs developmentally from single-polarity to dual-polarity coupling to facilitate the complex encoding of seasonal time in the system (Obrietan and Van den Pol, 1995; Myung et al., 2015).

The unique conditions in early development have clinical implications for preterm infant development in the neonatal intensive care unit (NICU) (Hazelhoff et al. in this collection). Light as the primary zeitgeber can impact perinatal development, and impairment in light entrainment has been implicated in an autism model mouse (Alamilla et al. in this collection). In this study, the authors found that Shank3^+/−^ mice (an animal model linked to autism, with a deletion in the ankyrin repeat domain) showed larger light pulse-induced phase shifts and increased VIP immunoreactivity in SCN neurons. One convincing explanation for preterm mortality in the NICU comes from the absence of the placenta-fetus platform where rich circadian endocrine exchanges occur (Astiz and Oster in this collection). As a demonstration of this principle, LuŽná et al. (in this collection) reported direct alterations to the molecular clock of the fetal SCN after maternal chrono-disruption. The LuŽná et al. study and that of Caba et al. (in this collection) hint at the role of feeding entrainment in early development. At the other end of the developmental spectrum, aging is associated with a decline in circadian timekeeping (Nakamura et al., 2011). This can be caused in part by age-related changes in single cell properties of the SCN (Ashimori et al.; Rezwana et al., both in this collection). Relevant to this point, circadian feeding entrainment appears to play a role in normalizing physiological outputs despite an aging circadian clock (Aoyama et al., in this collection).

The systematic changes in circadian clocks across the lifetime may be summarized by a schematic, which tracks the co-evolution of the single-cell molecular clock and the network (Figure 1). Intracellular events such as the TTFL and the brain network develop with different timing. The TTFL develops during the embryonic stage, but the network matures after birth. Although the transcriptional circuits of the circadian clock in individual cells are initially incomplete, they oscillate and synchronize through intercellular coupling in the perinatal phase. The high degree of synchronization in this stage is supported by the rhythmic availability of glucocorticoids, an exceptionally strong circadian clock resetting hormone. This signal is communicated embryonically *via* the placenta and postnatally *via* maternal milk, combined with a high expression of glucocorticoid receptors in these stages (Astiz and Oster). Each clock cell is a weak oscillator at these developmental time points, so they can be influenced by non-photic cues such as those derived from feeding. At maturation, the coupled circadian clock network is organized into a heterogeneous system through a diverse expression of peptides (Varadarajan et al., 2018). The robustness of the both cellular and network of clocks declines toward the end of life, and the clocks may again become more susceptible to feeding entrainment.

**Figure 1:**
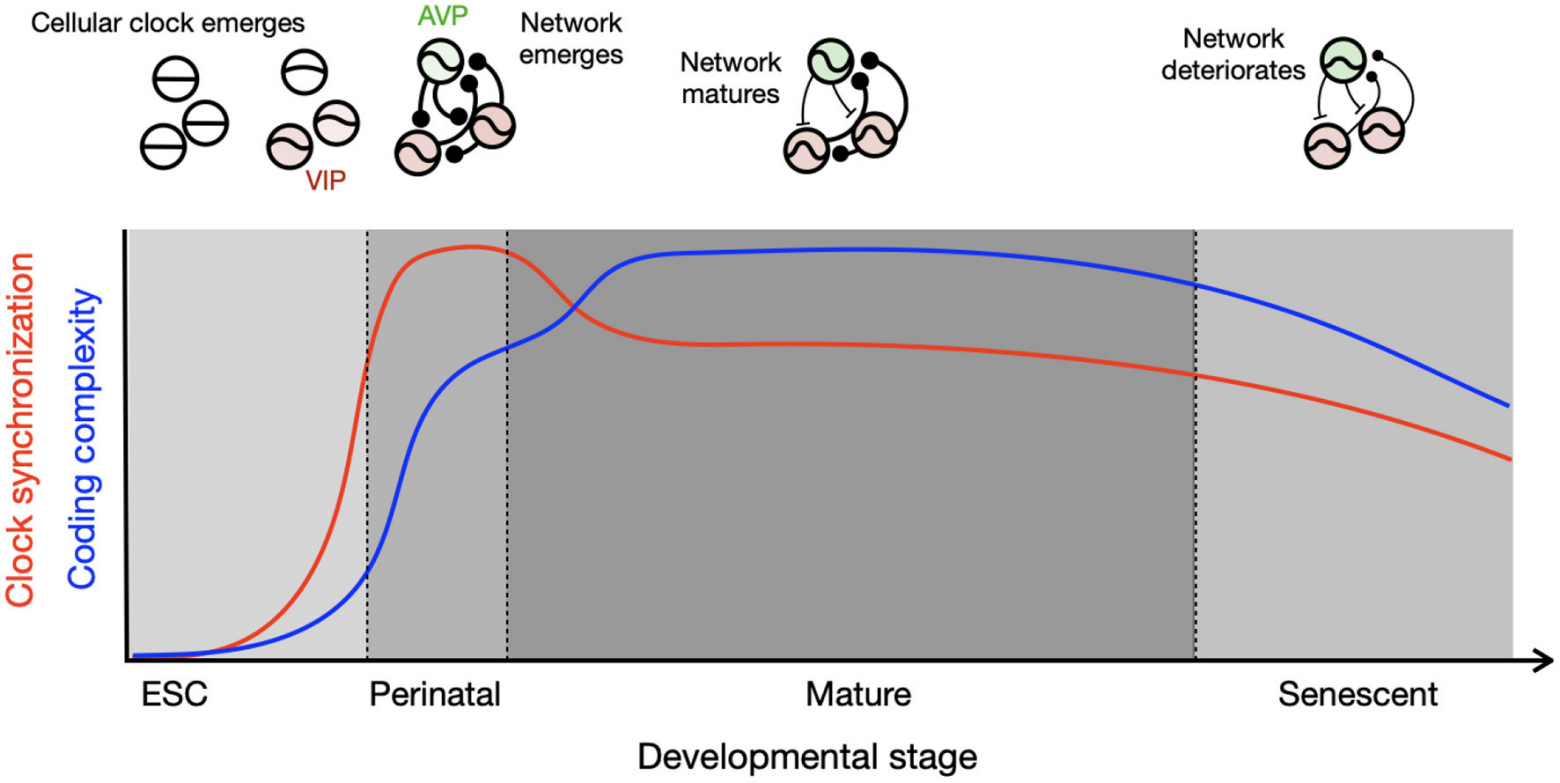
Development of cellular and network circadian clocks characterized by clock synchronization and complexity. Single-cell molecular clocks and their inter-cellular network evolve together through the course of development, which results in divergent parallel development of synchronization (red) and complexity (blue). The circadian clock emerges in single cells after embryonic stem cell (ESC) stage and connections are made in single positive polarity (round ending) in the perinatal stage pushed by homogeneously excitatory GABA and high expression of GR, resulting in strong synchronization among the clock cells. Diversity in neuropeptidergic expression and mixture of excitatory and inhibitory GABA (T-shaped ending) develop toward maturation, which slightly lowers the degree of synchronization but allows for complexity by a wider distribution of clock phases as found during seasonal time encoding. The schematics in the upper panel illustrate oscillatory properties of single cells and network coupling for each developmental stage, with thicker connection line indicating stronger coupling.

In summary, the work in this issue highlights the finding that the gradual modifications that occur in cells and networks among cells shape internal circadian homeostasis and hence the temporal boundaries of events in the brain and the rest of the body. Developmental changes throughout the life span direct the emergence and organization of circadian clocks, the clocks themselves have profound effects on development throughout life.

## Author Contributions

JM and DO wrote the manuscript with support of TJN, JRJ, and RS. All authors contributed to the article and approved the submitted version.

## Conflict of Interest

The authors declare that the research was conducted in the absence of any commercial or financial relationships that could be construed as a potential conflict of interest.

## Publisher's Note

All claims expressed in this article are solely those of the authors and do not necessarily represent those of their affiliated organizations, or those of the publisher, the editors and the reviewers. Any product that may be evaluated in this article, or claim that may be made by its manufacturer, is not guaranteed or endorsed by the publisher.
